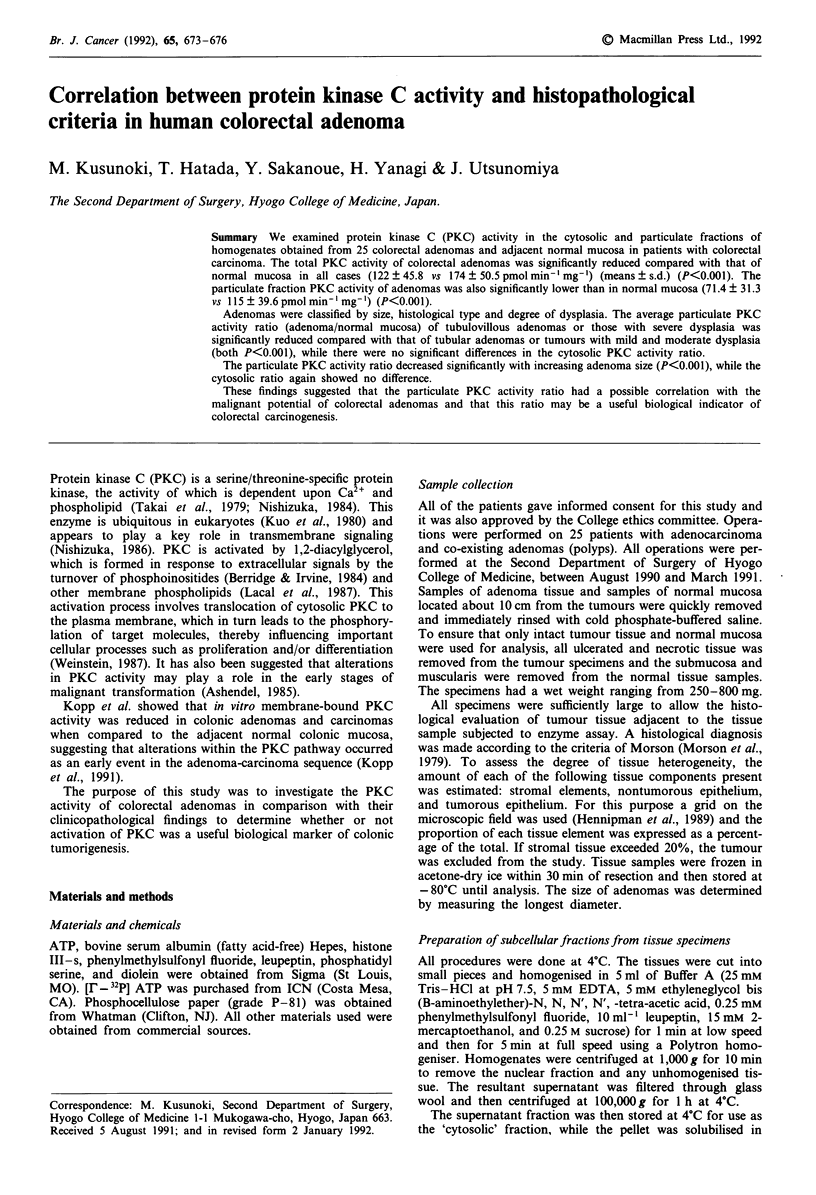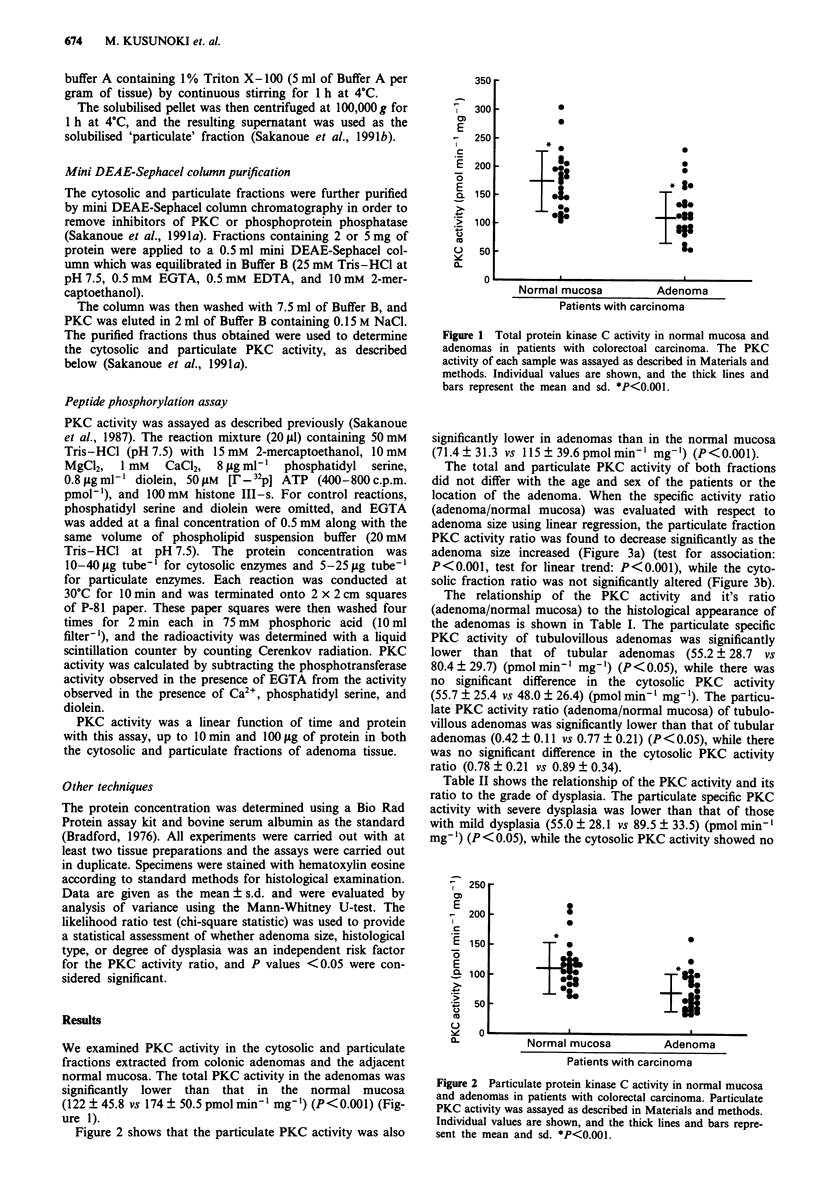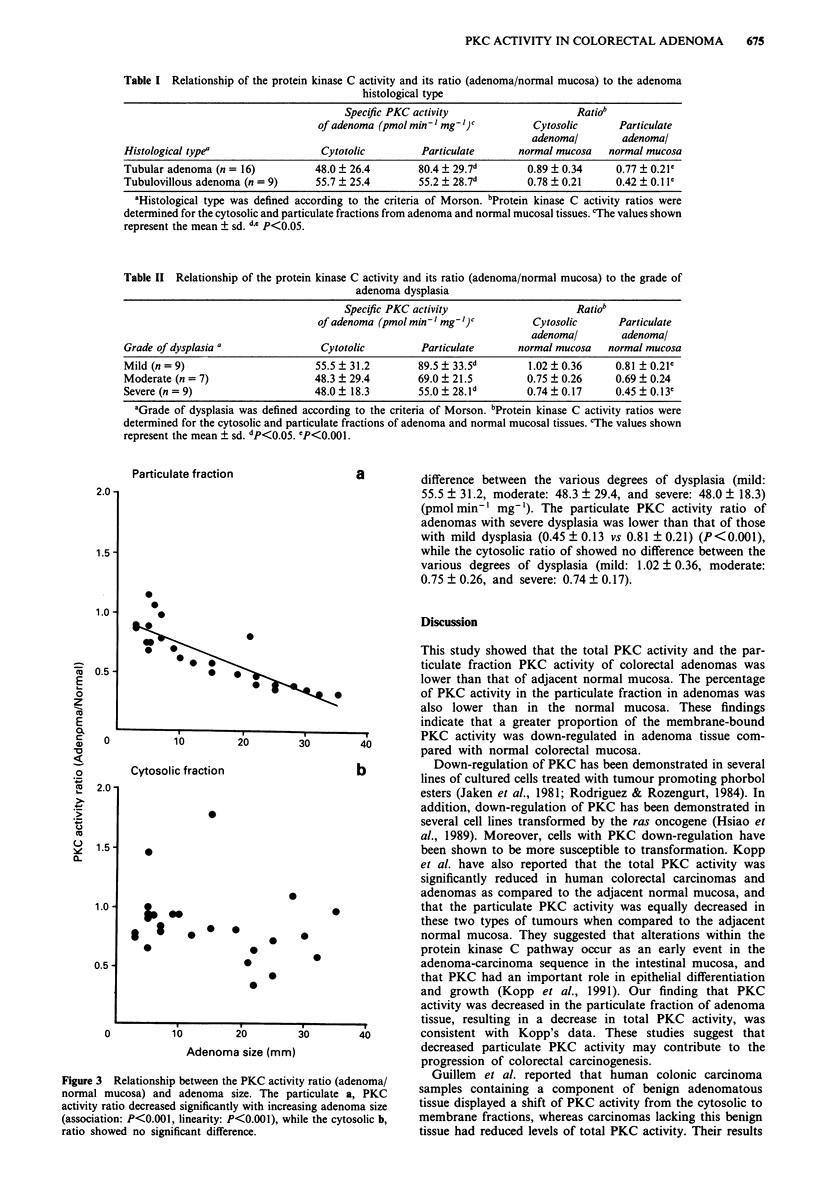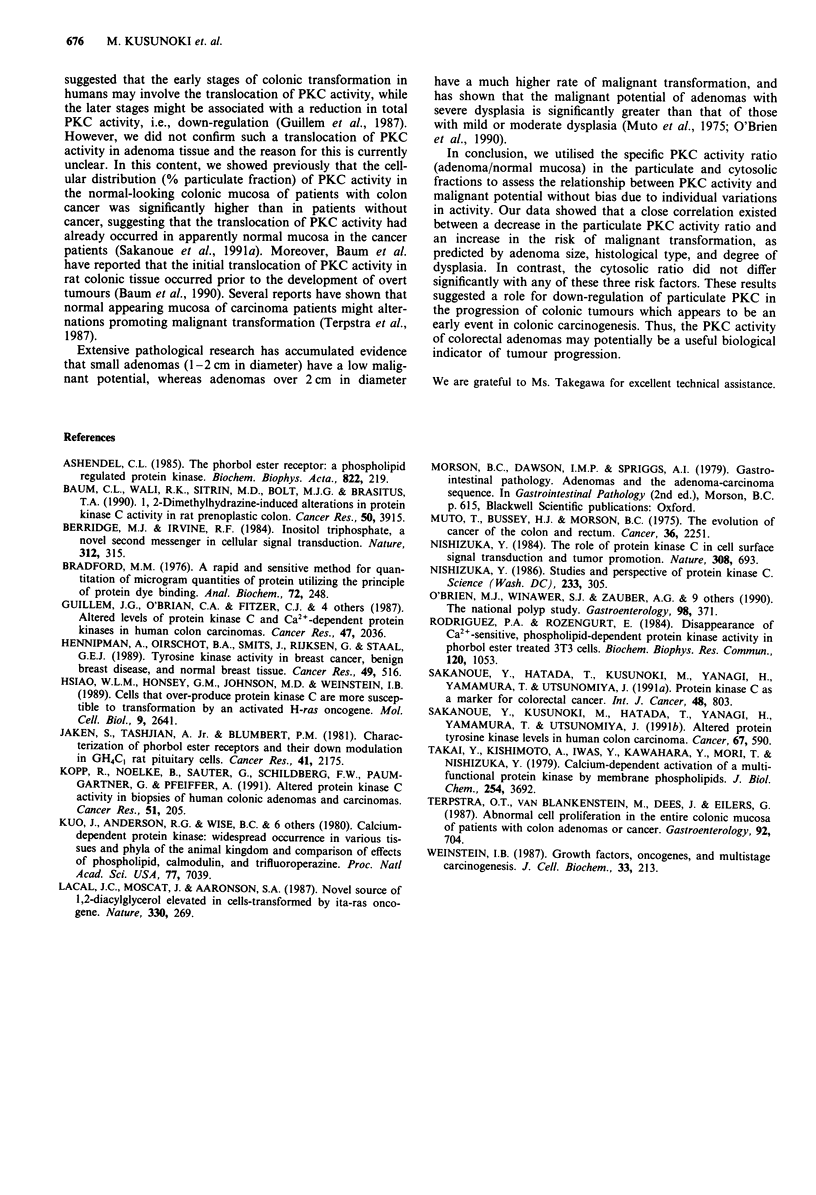# Correlation between protein kinase C activity and histopathological criteria in human colorectal adenoma.

**DOI:** 10.1038/bjc.1992.143

**Published:** 1992-05

**Authors:** M. Kusunoki, T. Hatada, Y. Sakanoue, H. Yanagi, J. Utsunomiya

**Affiliations:** Second Department of Surgery, Hyogo College of Medicine, Japan.

## Abstract

We examined protein kinase C (PKC) activity in the cytosolic and particulate fractions of homogenates obtained from 25 colorectal adenomas and adjacent normal mucosa in patients with colorectal carcinoma. The total PKC activity of colorectal adenomas was significantly reduced compared with that of normal mucosa in all cases (122 +/- 45.8 vs 174 +/- 50.5 pmol min-1 mg-1) (means +/- s.d.) (P less than 0.001). The particulate fraction PKC activity of adenomas was also significantly lower than in normal mucosa (71.4 +/- 31.3 vs 115 +/- 39.6 pmol min-1 mg-1) (P less than 0.001). Adenomas were classified by size, histological type and degree of dysplasia. The average particulate PKC activity ratio (adenoma/normal mucosa) of tubulovillous adenomas or those with severe dysplasia was significantly reduced compared with that of tubular adenomas or tumours with mild and moderate dysplasia (both P less than 0.001), while there were no significant differences in the cytosolic PKC activity ratio. The particulate PKC activity ratio decreased significantly with increasing adenoma size (P less than 0.001), while the cytosolic ratio again showed no difference. These findings suggested that the particulate PKC activity ratio had a possible correlation with the malignant potential of colorectal adenomas and that this ratio may be a useful biological indicator of colorectal carcinogenesis.


Br. J. Cancer (1992), 65, 673–676 © Macmillan Press Ltd., 1992

### Correlation between protein kinase C activity and histopathological criteria in human colorectal adenoma

M. Kusunoki, T. Hatada, Y. Sakanoue, H. Yanagi & J. Utsunomiya

*The Second Department of Surgery, Hyogo College of Medicine, Japan.*

**Summary** We examined protein kinase C (PKC) activity in the cytosolic and particulate fractions of homogenates obtained from 25 colorectal adenomas and adjacent normal mucosa in patients with colorectal carcinoma. The total PKC activity of colorectal adenomas was significantly reduced compared with that of normal mucosa in all cases ($122 \pm 45.8$ *vs* $174 \pm 50.5$ pmol $min^{-1}$ $mg^{-1}$) (means ± s.d.) ($P<0.001$). The particulate fraction PKC activity of adenomas was also significantly lower than in normal mucosa ($71.4 \pm 31.3$ *vs* $115 \pm 39.6$ pmol $min^{-1}$ $mg^{-1}$) ($P<0.001$).

Adenomas were classified by size, histological type and degree of dysplasia. The average particulate PKC activity ratio (adenoma/normal mucosa) of tubulovillous adenomas or those with severe dysplasia was significantly reduced compared with that of tubular adenomas or tumours with mild and moderate dysplasia (both $P<0.001$), while there were no significant differences in the cytosolic PKC activity ratio.

The particulate PKC activity ratio decreased significantly with increasing adenoma size ($P<0.001$), while the cytosolic ratio again showed no difference.

These findings suggested that the particulate PKC activity ratio had a possible correlation with the malignant potential of colorectal adenomas and that this ratio may be a useful biological indicator of colorectal carcinogenesis.

Protein kinase C (PKC) is a serine/threonine-specific protein kinase, the activity of which is dependent upon $Ca^{2+}$ and phospholipid (Takai *et al.*, 1979; Nishizuka, 1984). This enzyme is ubiquitous in eukaryotes (Kuo *et al.*, 1980) and appears to play a key role in transmembrane signaling (Nishizuka, 1986). PKC is activated by 1,2-diacylglycerol, which is formed in response to extracellular signals by the turnover of phosphoinositides (Berridge & Irvine, 1984) and other membrane phospholipids (Lacal *et al.*, 1987). This activation process involves translocation of cytosolic PKC to the plasma membrane, which in turn leads to the phosphorylation of target molecules, thereby influencing important cellular processes such as proliferation and/or differentiation (Weinstein, 1987). It has also been suggested that alterations in PKC activity may play a role in the early stages of malignant transformation (Ashendel, 1985).

Kopp *et al.* showed that *in vitro* membrane-bound PKC activity was reduced in colonic adenomas and carcinomas when compared to the adjacent normal colonic mucosa, suggesting that alterations within the PKC pathway occurred as an early event in the adenoma-carcinoma sequence (Kopp *et al.*, 1991).

The purpose of this study was to investigate the PKC activity of colorectal adenomas in comparison with their clinicopathological findings to determine whether or not activation of PKC was a useful biological marker of colonic tumorigenesis.

### Materials and methods

### *Materials and chemicals*

ATP, bovine serum albumin (fatty acid-free) Hepes, histone III-s, phenylmethylsulfonyl fluoride, leupeptin, phosphatidyl serine, and diolein were obtained from Sigma (St Louis, MO). [$\Gamma - ^{32}P$] ATP was purchased from ICN (Costa Mesa, CA). Phosphocellulose paper (grade P-81) was obtained from Whatman (Clifton, NJ). All other materials used were obtained from commercial sources.

### *Sample collection*

All of the patients gave informed consent for this study and it was also approved by the College ethics committee. Operations were performed on 25 patients with adenocarcinoma and co-existing adenomas (polyps). All operations were performed at the Second Department of Surgery of Hyogo College of Medicine, between August 1990 and March 1991. Samples of adenoma tissue and samples of normal mucosa located about 10 cm from the tumours were quickly removed and immediately rinsed with cold phosphate-buffered saline. To ensure that only intact tumour tissue and normal mucosa were used for analysis, all ulcerated and necrotic tissue was removed from the tumour specimens and the submucosa and muscularis were removed from the normal tissue samples. The specimens had a wet weight ranging from 250–800 mg.

All specimens were sufficiently large to allow the histological evaluation of tumour tissue adjacent to the tissue sample subjected to enzyme assay. A histological diagnosis was made according to the criteria of Morson (Morson *et al.*, 1979). To assess the degree of tissue heterogeneity, the amount of each of the following tissue components present was estimated: stromal elements, nontumorous epithelium, and tumorous epithelium. For this purpose a grid on the microscopic field was used (Hennipman *et al.*, 1989) and the proportion of each tissue element was expressed as a percentage of the total. If stromal tissue exceeded 20%, the tumour was excluded from the study. Tissue samples were frozen in acetone-dry ice within 30 min of resection and then stored at −80°C until analysis. The size of adenomas was determined by measuring the longest diameter.

### *Preparation of subcellular fractions from tissue specimens*

All procedures were done at 4°C. The tissues were cut into small pieces and homogenised in 5 ml of Buffer A (25 mM Tris-HCl at pH 7.5, 5 mM EDTA, 5 mM ethyleneglycol bis (B-aminoethylether)-N, N, N′, N′, -tetra-acetic acid, 0.25 mM phenylmethylsulfonyl fluoride, 10 $ml^{-1}$ leupeptin, 15 mM 2-mercaptoethanol, and 0.25 M sucrose) for 1 min at low speed and then for 5 min at full speed using a Polytron homogeniser. Homogenates were centrifuged at 1,000 *g* for 10 min to remove the nuclear fraction and any unhomogenised tissue. The resultant supernatant was filtered through glass wool and then centrifuged at 100,000 *g* for 1 h at 4°C.

The supernatant fraction was then stored at 4°C for use as the 'cytosolic' fraction, while the pellet was solubilised in

Correspondence: M. Kusunoki, Second Department of Surgery, Hyogo College of Medicine 1-1 Mukogawa-cho, Hyogo, Japan 663.
Received 5 August 1991; and in revised form 2 January 1992.

buffer A containing 1% Triton X-100 (5 ml of Buffer A per gram of tissue) by continuous stirring for 1 h at 4°C.

The solubilised pellet was then centrifuged at 100,000 *g* for 1 h at 4°C, and the resulting supernatant was used as the solubilised 'particulate' fraction (Sakanoue *et al.*, 1991*b*).

*Mini DEAE-Sephacel column purification*

The cytosolic and particulate fractions were further purified by mini DEAE-Sephacel column chromatography in order to remove inhibitors of PKC or phosphoprotein phosphatase (Sakanoue *et al.*, 1991*a*). Fractions containing 2 or 5 mg of protein were applied to a 0.5 ml mini DEAE-Sephacel column which was equilibrated in Buffer B (25 mM Tris-HCl at pH 7.5, 0.5 mM EGTA, 0.5 mM EDTA, and 10 mM 2-mercaptoethanol).

The column was then washed with 7.5 ml of Buffer B, and PKC was eluted in 2 ml of Buffer B containing 0.15 M NaCl. The purified fractions thus obtained were used to determine the cytosolic and particulate PKC activity, as described below (Sakanoue *et al.*, 1991*a*).

*Peptide phosphorylation assay*

PKC activity was assayed as described previously (Sakanoue *et al.*, 1987). The reaction mixture (20 µl) containing 50 mM Tris-HCl (pH 7.5) with 15 mM 2-mercaptoethanol, 10 mM $MgCl_2$, 1 mM $CaCl_2$, 8 µg $ml^{-1}$ phosphatidyl serine, 0.8 µg $ml^{-1}$ diolein, 50 µM [Γ-$^{32}$p] ATP (400-800 c.p.m. $pmol^{-1}$), and 100 mM histone III-s. For control reactions, phosphatidyl serine and diolein were omitted, and EGTA was added at a final concentration of 0.5 mM along with the same volume of phospholipid suspension buffer (20 mM Tris-HCl at pH 7.5). The protein concentration was 10-40 µg $tube^{-1}$ for cytosolic enzymes and 5-25 µg $tube^{-1}$ for particulate enzymes. Each reaction was conducted at 30°C for 10 min and was terminated onto 2 × 2 cm squares of P-81 paper. These paper squares were then washed four times for 2 min each in 75 mM phosphoric acid (10 ml $filter^{-1}$), and the radioactivity was determined with a liquid scintillation counter by counting Cerenkov radiation. PKC activity was calculated by subtracting the phosphotransferase activity observed in the presence of EGTA from the activity observed in the presence of $Ca^{2+}$, phosphatidyl serine, and diolein.

PKC activity was a linear function of time and protein with this assay, up to 10 min and 100 µg of protein in both the cytosolic and particulate fractions of adenoma tissue.

*Other techniques*

The protein concentration was determined using a Bio Rad Protein assay kit and bovine serum albumin as the standard (Bradford, 1976). All experiments were carried out with at least two tissue preparations and the assays were carried out in duplicate. Specimens were stained with hematoxylin eosine according to standard methods for histological examination. Data are given as the mean ± s.d. and were evaluated by analysis of variance using the Mann-Whitney U-test. The likelihood ratio test (chi-square statistic) was used to provide a statistical assessment of whether adenoma size, histological type, or degree of dysplasia was an independent risk factor for the PKC activity ratio, and *P* values <0.05 were considered significant.

### Results

We examined PKC activity in the cytosolic and particulate fractions extracted from colonic adenomas and the adjacent normal mucosa. The total PKC activity in the adenomas was significantly lower than that in the normal mucosa (122 ± 45.8 *vs* 174 ± 50.5 pmol $min^{-1}$ $mg^{-1}$) ($P<0.001$) (Figure 1).

Figure 2 shows that the particulate PKC activity was also significantly lower in adenomas than in the normal mucosa (71.4 ± 31.3 *vs* 115 ± 39.6 pmol $min^{-1}$ $mg^{-1}$) ($P<0.001$).

**Figure 1** Total protein kinase C activity in normal mucosa and adenomas in patients with colorectoal carcinoma. The PKC activity of each sample was assayed as described in Materials and methods. Individual values are shown, and the thick lines and bars represent the mean and sd. *$P<0.001$.

The total and particulate PKC activity of both fractions did not differ with the age and sex of the patients or the location of the adenoma. When the specific activity ratio (adenoma/normal mucosa) was evaluated with respect to adenoma size using linear regression, the particulate fraction PKC activity ratio was found to decrease significantly as the adenoma size increased (Figure 3a) (test for association: $P<0.001$, test for linear trend: $P<0.001$), while the cytosolic fraction ratio was not significantly altered (Figure 3b).

The relationship of the PKC activity and it's ratio (adenoma/normal mucosa) to the histological appearance of the adenomas is shown in Table I. The particulate specific PKC activity of tubulovillous adenomas was significantly lower than that of tubular adenomas (55.2 ± 28.7 *vs* 80.4 ± 29.7) (pmol $min^{-1}$ $mg^{-1}$) ($P<0.05$), while there was no significant difference in the cytosolic PKC activity (55.7 ± 25.4 *vs* 48.0 ± 26.4) (pmol $min^{-1}$ $mg^{-1}$). The particulate PKC activity ratio (adenoma/normal mucosa) of tubulovillous adenomas was significantly lower than that of tubular adenomas (0.42 ± 0.11 *vs* 0.77 ± 0.21) ($P<0.05$), while there was no significant difference in the cytosolic PKC activity ratio (0.78 ± 0.21 *vs* 0.89 ± 0.34).

Table II shows the relationship of the PKC activity and its ratio to the grade of dysplasia. The particulate specific PKC activity with severe dysplasia was lower than that of those with mild dysplasia (55.0 ± 28.1 *vs* 89.5 ± 33.5) (pmol $min^{-1}$ $mg^{-1}$) ($P<0.05$), while the cytosolic PKC activity showed no

**Figure 2** Particulate protein kinase C activity in normal mucosa and adenomas in patients with colorectal carcinoma. Particulate PKC activity was assayed as described in Materials and methods. Individual values are shown, and the thick lines and bars represent the mean and sd. *$P<0.001$.

**Table I** Relationship of the protein kinase C activity and its ratio (adenoma/normal mucosa) to the adenoma histological type

| | *Specific PKC activity of adenoma (pmol min$^{-1}$ mg$^{-1}$)*[c] | | *Ratio*[b] | |
|---|---|---|---|---|
| *Histological type*[a] | *Cytotolic* | *Particulate* | *Cytosolic adenoma/ normal mucosa* | *Particulate adenoma/ normal mucosa* |
| Tubular adenoma ($n$ = 16) | 48.0 ± 26.4 | 80.4 ± 29.7[d] | 0.89 ± 0.34 | 0.77 ± 0.21[e] |
| Tubulovillous adenoma ($n$ = 9) | 55.7 ± 25.4 | 55.2 ± 28.7[d] | 0.78 ± 0.21 | 0.42 ± 0.11[e] |

[a]Histological type was defined according to the criteria of Morson. [b]Protein kinase C activity ratios were determined for the cytosolic and particulate fractions from adenoma and normal mucosal tissues. [c]The values shown represent the mean ± sd. [d,e] $P<0.05$.

**Table II** Relationship of the protein kinase C activity and its ratio (adenoma/normal mucosa) to the grade of adenoma dysplasia

| | *Specific PKC activity of adenoma (pmol min$^{-1}$ mg$^{-1}$)*[c] | | *Ratio*[b] | |
|---|---|---|---|---|
| *Grade of dysplasia* [a] | *Cytotolic* | *Particulate* | *Cytosolic adenoma/ normal mucosa* | *Particulate adenoma/ normal mucosa* |
| Mild ($n$ = 9) | 55.5 ± 31.2 | 89.5 ± 33.5[d] | 1.02 ± 0.36 | 0.81 ± 0.21[e] |
| Moderate ($n$ = 7) | 48.3 ± 29.4 | 69.0 ± 21.5 | 0.75 ± 0.26 | 0.69 ± 0.24 |
| Severe ($n$ = 9) | 48.0 ± 18.3 | 55.0 ± 28.1[d] | 0.74 ± 0.17 | 0.45 ± 0.13[e] |

[a]Grade of dysplasia was defined according to the criteria of Morson. [b]Protein kinase C activity ratios were determined for the cytosolic and particulate fractions of adenoma and normal mucosal tissues. [c]The values shown represent the mean ± sd. [d]$P<0.05$. [e]$P<0.001$.

**Figure 3** Relationship between the PKC activity ratio (adenoma/normal mucosa) and adenoma size. The particulate **a**, PKC activity ratio decreased significantly with increasing adenoma size (association: $P<0.001$, linearity: $P<0.001$), while the cytosolic **b**, ratio showed no significant difference.

difference between the various degrees of dysplasia (mild: 55.5 ± 31.2, moderate: 48.3 ± 29.4, and severe: 48.0 ± 18.3) (pmol min$^{-1}$ mg$^{-1}$). The particulate PKC activity ratio of adenomas with severe dysplasia was lower than that of those with mild dysplasia (0.45 ± 0.13 *vs* 0.81 ± 0.21) ($P<0.001$), while the cytosolic ratio of showed no difference between the various degrees of dysplasia (mild: 1.02 ± 0.36, moderate: 0.75 ± 0.26, and severe: 0.74 ± 0.17).

### Discussion

This study showed that the total PKC activity and the particulate fraction PKC activity of colorectal adenomas was lower than that of adjacent normal mucosa. The percentage of PKC activity in the particulate fraction in adenomas was also lower than in the normal mucosa. These findings indicate that a greater proportion of the membrane-bound PKC activity was down-regulated in adenoma tissue compared with normal colorectal mucosa.

Down-regulation of PKC has been demonstrated in several lines of cultured cells treated with tumour promoting phorbol esters (Jaken *et al.*, 1981; Rodriguez & Rozengurt, 1984). In addition, down-regulation of PKC has been demonstrated in several cell lines transformed by the *ras* oncogene (Hsiao *et al.*, 1989). Moreover, cells with PKC down-regulation have been shown to be more susceptible to transformation. Kopp *et al.* have also reported that the total PKC activity was significantly reduced in human colorectal carcinomas and adenomas as compared to the adjacent normal mucosa, and that the particulate PKC activity was equally decreased in these two types of tumours when compared to the adjacent normal mucosa. They suggested that alterations within the protein kinase C pathway occur as an early event in the adenoma-carcinoma sequence in the intestinal mucosa, and that PKC had an important role in epithelial differentiation and growth (Kopp *et al.*, 1991). Our finding that PKC activity was decreased in the particulate fraction of adenoma tissue, resulting in a decrease in total PKC activity, was consistent with Kopp's data. These studies suggest that decreased particulate PKC activity may contribute to the progression of colorectal carcinogenesis.

Guillem *et al.* reported that human colonic carcinoma samples containing a component of benign adenomatous tissue displayed a shift of PKC activity from the cytosolic to membrane fractions, whereas carcinomas lacking this benign tissue had reduced levels of total PKC activity. Their results suggested that the early stages of colonic transformation in humans may involve the translocation of PKC activity, while the later stages might be associated with a reduction in total PKC activity, i.e., down-regulation (Guillem *et al.*, 1987). However, we did not confirm such a translocation of PKC activity in adenoma tissue and the reason for this is currently unclear. In this content, we showed previously that the cellular distribution (% particulate fraction) of PKC activity in the normal-looking colonic mucosa of patients with colon cancer was significantly higher than in patients without cancer, suggesting that the translocation of PKC activity had already occurred in apparently normal mucosa in the cancer patients (Sakanoue *et al.*, 1991*a*). Moreover, Baum *et al.* have reported that the initial translocation of PKC activity in rat colonic tissue occurred prior to the development of overt tumours (Baum *et al.*, 1990). Several reports have shown that normal appearing mucosa of carcinoma patients might alternations promoting malignant transformation (Terpstra *et al.*, 1987).

Extensive pathological research has accumulated evidence that small adenomas (1–2 cm in diameter) have a low malignant potential, whereas adenomas over 2 cm in diameter have a much higher rate of malignant transformation, and has shown that the malignant potential of adenomas with severe dysplasia is significantly greater than that of those with mild or moderate dysplasia (Muto *et al.*, 1975; O'Brien *et al.*, 1990).

In conclusion, we utilised the specific PKC activity ratio (adenoma/normal mucosa) in the particulate and cytosolic fractions to assess the relationship between PKC activity and malignant potential without bias due to individual variations in activity. Our data showed that a close correlation existed between a decrease in the particulate PKC activity ratio and an increase in the risk of malignant transformation, as predicted by adenoma size, histological type, and degree of dysplasia. In contrast, the cytosolic ratio did not differ significantly with any of these three risk factors. These results suggested a role for down-regulation of particulate PKC in the progression of colonic tumours which appears to be an early event in colonic carcinogenesis. Thus, the PKC activity of colorectal adenomas may potentially be a useful biological indicator of tumour progression.

We are grateful to Ms. Takegawa for excellent technical assistance.

### References

ASHENDEL, C.L. (1985). The phorbol ester receptor: a phospholipid regulated protein kinase. *Biochem. Biophys. Acta.*, **822**, 219.

BAUM, C.L., WALI, R.K., SITRIN, M.D., BOLT, M.J.G. & BRASITUS, T.A. (1990). 1, 2-Dimethylhydrazine-induced alterations in protein kinase C activity in rat prenoplastic colon. *Cancer Res.*, **50**, 3915.

BERRIDGE, M.J. & IRVINE, R.F. (1984). Inositol triphosphate, a novel second messenger in cellular signal transduction. *Nature*, **312**, 315.

BRADFORD, M.M. (1976). A rapid and sensitive method for quantitation of microgram quantities of protein utilizing the principle of protein dye binding. *Anal. Biochem.*, **72**, 248.

GUILLEM, J.G., O'BRIAN, C.A. & FITZER, C.J. & 4 others (1987). Altered levels of protein kinase C and $Ca^{2+}$-dependent protein kinases in human colon carcinomas. *Cancer Res.*, **47**, 2036.

HENNIPMAN, A., OIRSCHOT, B.A., SMITS, J., RIJKSEN, G. & STAAL, G.E.J. (1989). Tyrosine kinase activity in breast cancer, benign breast disease, and normal breast tissue. *Cancer Res.*, **49**, 516.

HSIAO, W.L.M., HONSEY, G.M., JOHNSON, M.D. & WEINSTEIN, I.B. (1989). Cells that over-produce protein kinase C are more susceptible to transformation by an activated H-*ras* oncogene. *Mol. Cell. Biol.*, **9**, 2641.

JAKEN, S., TASHJIAN, A. Jr. & BLUMBERT, P.M. (1981). Characterization of phorbol ester receptors and their down modulation in $GH_4C_1$ rat pituitary cells. *Cancer Res.*, **41**, 2175.

KOPP, R., NOELKE, B., SAUTER, G., SCHILDBERG, F.W., PAUMGARTNER, G. & PFEIFFER, A. (1991). Altered protein kinase C activity in biopsies of human colonic adenomas and carcinomas. *Cancer Res.*, **51**, 205.

KUO, J., ANDERSON, R.G. & WISE, B.C. & 6 others (1980). Calcium-dependent protein kinase: widespread occurrence in various tissues and phyla of the animal kingdom and comparison of effects of phospholipid, calmodulin, and trifluoroperazine. *Proc. Natl Acad. Sci. USA*, **77**, 7039.

LACAL, J.C., MOSCAT, J. & AARONSON, S.A. (1987). Novel source of 1,2-diacylglycerol elevated in cells-transformed by ita-ras oncogene. *Nature*, **330**, 269.

MORSON, B.C., DAWSON, I.M.P. & SPRIGGS, A.I. (1979). Gastrointestinal pathology. Adenomas and the adenoma-carcinoma sequence. In *Gastrointestinal Pathology* (2nd ed.), Morson, B.C. p. 615, Blackwell Scientific publications: Oxford.

MUTO, T., BUSSEY, H.J. & MORSON, B.C. (1975). The evolution of cancer of the colon and rectum. *Cancer*, **36**, 2251.

NISHIZUKA, Y. (1984). The role of protein kinase C in cell surface signal transduction and tumor promotion. *Nature*, **308**, 693.

NISHIZUKA, Y. (1986). Studies and perspective of protein kinase C. *Science (Wash. DC)*, **233**, 305.

O'BRIEN, M.J., WINAWER, S.J. & ZAUBER, A.G. & 9 others (1990). The national polyp study. *Gastroenterology*, **98**, 371.

RODRIGUEZ, P.A. & ROZENGURT, E. (1984). Disappearance of $Ca^{2+}$-sensitive, phospholipid-dependent protein kinase activity in phorbol ester treated 3T3 cells. *Biochem. Biophys. Res. Commun.*, **120**, 1053.

SAKANOUE, Y., HATADA, T., KUSUNOKI, M., YANAGI, H., YAMAMURA, T. & UTSUNOMIYA, J. (1991*a*). Protein kinase C as a marker for colorectal cancer. *Int. J. Cancer*, **48**, 803.

SAKANOUE, Y., KUSUNOKI, M., HATADA, T., YANAGI, H., YAMAMURA, T. & UTSUNOMIYA, J. (1991*b*). Altered protein tyrosine kinase levels in human colon carcinoma. *Cancer*, **67**, 590.

TAKAI, Y., KISHIMOTO, A., IWAS, Y., KAWAHARA, Y., MORI, T. & NISHIZUKA, Y. (1979). Calcium-dependent activation of a multifunctional protein kinase by membrane phospholipids. *J. Biol. Chem.*, **254**, 3692.

TERPSTRA, O.T., VAN BLANKENSTEIN, M., DEES, J. & EILERS, G. (1987). Abnormal cell proliferation in the entire colonic mucosa of patients with colon adenomas or cancer. *Gastroenterology*, **92**, 704.

WEINSTEIN, I.B. (1987). Growth factors, oncogenes, and multistage carcinogenesis. *J. Cell. Biochem.*, **33**, 213.